# Retraction: Occurrence of L1014F and L1014S mutations in insecticide resistant *Culex quinquefasciatus* from filariasis endemic districts of West Bengal, India

**DOI:** 10.1371/journal.pntd.0011417

**Published:** 2023-06-20

**Authors:** The PLOS Neglected Tropical Diseases Editors

After this article [1] was published, concerns were raised about the Supporting Information files. Specifically:

- In S1 Fig, there is a region at the top of lanes 3–5 where the background does not appear to match the overall background of the figure, and where horizontal and vertical discontinuities are present.
- In S2 Fig, the DNA sequence chromatograms have been provided to confirm the presence of three alleles at the L1014 position of the voltage-gated sodium channel gene but do not appear to include 1014 codons.
- The DNA sequence patterns for panels A and B in S2 Fig appear to be the same.

In response to queries about these experiments, the first author provided the original uncropped and unadjusted image file captured at the time of the experiment for S1 Fig, and stated that the figure was edited for proper visualization of the bands and to avoid smearing in the gel.

During follow-up discussions, the first author requested retraction of this article [1] due to an error in the interpretation of the sequencing data. The first author stated that instead of studying the 122 bp (for forward primer, Cgd1) and 147–148 bp (for reverse primer, Cgd2) region of the sequence, they highlighted the incorrect region of the sequence near poly A region. The first author also stated that L1014 (TTA) and L1014F (TTT) is present at 122 bp when using forward primer flanked by AAT and GTC which proves the presence of L1014F mutation in the study. However, they also stated that L1014S mutation is neither present in 122 bp (forward primer, Cgd1) nor at 147–148 bp (reverse primer, Cgd2) and that the absence of L1014S mutation impacts the results, discussion, and conclusions of this article [1].

In light of the above concerns, the conclusions are no longer supported and *PLOS Neglected Tropical Diseases* retracts this article. The editors regret that these concerns were not addressed prior to the article’s publication.

PR agreed with the retraction. DS either did not respond directly or could not be reached.
